# ﻿*Primulajiangyouensis* (Primulaceae), a new species of Primula sect. Auganthus from Sichuan, China

**DOI:** 10.3897/phytokeys.260.158039

**Published:** 2025-07-15

**Authors:** Jiu-lin Gu, Hua-dong Wang, Hong-qiang Lin, Xiao-qi Jiang, Tian Shuai, Zhi-kun Wu

**Affiliations:** 1 Department of Pharmacy, Guizhou University of Traditional Chinese Medicine, Guiyang, 550025, Guizhou, China Guizhou University of Traditional Chinese Medicine Guiyang China; 2 Sichuan College of Traditional Chinese Medicine, Mianyang, 621000, Sichuan, China Sichuan College of Traditional Chinese Medicine Mianyang China; 3 Sichuan Wolong National Natural Reserve Administration Bureau, Wenchuan, 623006, Sichuan, China Sichuan Wolong National Natural Reserve Administration Bureau Wenchuan China

**Keywords:** Conservation status, diversity, jiang you bao chun, taxonomy

## Abstract

*Primulajiangyouensis* J.L.Gu & Z.K.Wu, a new species of Primulaceae from Sichuan, China, is described and illustrated. Morphological evidence supports *P.jiangyouensis* as a member of P.sect.Auganthus, which is characterized by shallowly to deeply lobed leaves covered with hairs, and distinctively broad and flat-bottomed calyx. The new species is characterized by its branched and stout rhizomes usually up to 40 cm, yellow corollas with a distinct fan-shaped reddish-brown blotch at the base of lobes, and short glandular hair on aboveground parts. The distribution, morphological comparison with close related species and conservation status of the new species, as well as a key to the species of Primulasect.Auganthus, are also provided.

## ﻿Introduction

*Primula* L. represents one of the largest genera within the family Primulaceae, comprising approximately 589 species globally (POWO 2025). The genus exhibits a predominantly Holarctic distribution, with the majority of species inhabiting temperate and alpine zones of the Northern Hemisphere, while a limited number occur in the Southern Hemisphere (Hu 1990, 1994; Richards 2002). The southwestern region of China, particularly the Himalayan-Hengduan Mountains, serves as the primary center of diversity for *Primula*, harboring more than 300 species. These are concentrated chiefly in western Sichuan, eastern Xizang (Tibet), and northwestern Yunnan (Hu 1990; Hu and Kelso 1996; Richards 2002).

PrimulaSect.Auganthus (Link) Pax ex Balf. f. (39: 139, 1913)(Balfour 1913) is a small group within the genus *Primula*. It was recognized in “Flora Of China” as comprising three species: *P.filchnerae* Knuth, *P.sinensis* Sabine ex Lindley, and *P.rupestris* Balf. f. & Farrer (Hu and Kelso 1996). In 2023, an additional species, *P.xingshanensis* Y. B. Wang, was newly described, bringing the total number of accepted species in this section to four. These plants are primarily distributed in the border regions of Hubei, Shaanxi, and Sichuan in north-central China. The morphological characteristics of this section include shallowly to deeply lobed leaves covered with hairs, an inflated calyx with a globose or flattened base that does not tightly clasp the corolla tube, and a constricted upper part of the calyx that fits closely around the corolla tube.

As a biodiversity hotspot in China, Sichuan harbors approximately 170 species of *Primula* (Primulaceae) distributed across the region (Wu 1999). With intensified botanical exploration, numerous new *Primula* species have been documented in Sichuan over the past two decades (Hu and Ceng 2003; Hu and Hao 2006; Wu et al. 2013; Xu et al. 2014, 2015, 2016a, 2016b, 2016c, 2017a, 2017b, 2019; Ju et al. 2018, 2021, 2023; Yuan et al. 2018; Li et al. 2023; Luo et al. 2023, Li et al. 2024, Shuai et al. 2025).

During a botanical expedition to Jiangyou, Sichuan Province, southwestern China in November 2022, we encountered a distinctive population of *Primula* exhibiting unique morphological characteristics. The plants possessed lobed leaves densely covered with hair and an inflated calyx with a broad, flattened base, which are features characteristic of P.Sect.Auganthus. However, the plant displayed unusual floral morphology: branched and stout rhizomes usually up to 40 cm, yellow corollas with a distinct fan-shaped reddish-brown blotch at the base of lobes, and oblong-ovate leaf blades bearing short glandular hair. To thoroughly document this population, we conducted follow-up surveys of the type locality and adjacent areas in January 2023 and April 2024, collecting voucher specimens for detailed examination. Through comprehensive morphological analysis and comparison with relevant literature and herbarium specimens of related taxa, we concluded that this entity represents a previously undescribed species. The combination of diagnostic characters, including stout rhizomes, corolla morphology, indumentum features, and calyx structure, distinguishes this taxon from all known *Primula* species. We therefore formally describe it here as a new species, providing detailed morphological descriptions and illustrations.

## ﻿Materials and methods

Morphological observations, measurements, and descriptions (including habit, rhizome, hair, leaf, calyx, corolla, flower color, etc.) of the new species were conducted using both dried specimens and living plants collected from Jiangyou County, Sichuan Province. Comparative morphological analyses with closely related species were performed by examining specimens from major herbaria, such as PE, P, E, IBSC and KUN, as well as relevant literature (Smith and Fletcher 1946; Hu 1990; Halda 1992; Hu and Kelso 1996; Richards 2002; Wang et al. 2023). Key morphological characters of *P.jiangyouensis* and its allied species in P.sect.Auganthus, including *P.filchnerae*, *P.sinensis*, *P.rupestris*, and *P.xingshanensis*, were measured using a Vernier calliper on living plants from their type localities. The conservation status of the new species was evaluated following the guidelines of the IUCN Red List categories and criteria (IUCN Standards and Petitions Committee 2024). To facilitate the identification of P.sect.Auguanthus, a key to the species within this section, based on the primary morphological differences among these five species, was conducted.

## ﻿Taxonomic treatment

### ﻿ Primula jiangyouensis

Taxon classificationPlantaeEricalesPrimulaceae

J.L.Gu & Z.K.Wu sp. nov.

B43CE9C4-D3B0-5D29-8B1A-CF89932C6ED6

urn:lsid:ipni.org:names:77365615-1

#### Diagnosis.

**Figure 1.**
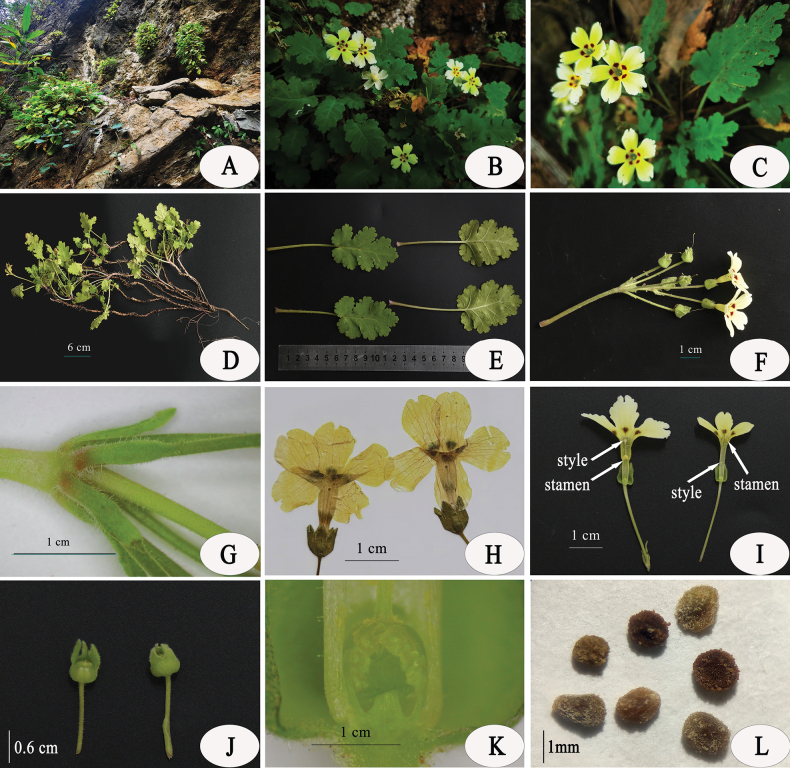
*Primulajiangyouensis* sp. nov. **A.** Habitat; **B, C.** Flowering plants; **D.** Plant with roots and rhizomes; **E.** Leaves, left: upper surface, right: lower surface; **F.** Infructescences; **G.** Bracts and pedicels; **H.** Flower, lateral view; **I.** Longitudinal section of pin flower (left) and thrum flower (right); **J.** Calyx and young fruit; **K.** Longitudinal section of ovary; **L.** Ripe seed. Photographed by J.L.Gu and Z.K.Wu.

**Figure 2.**
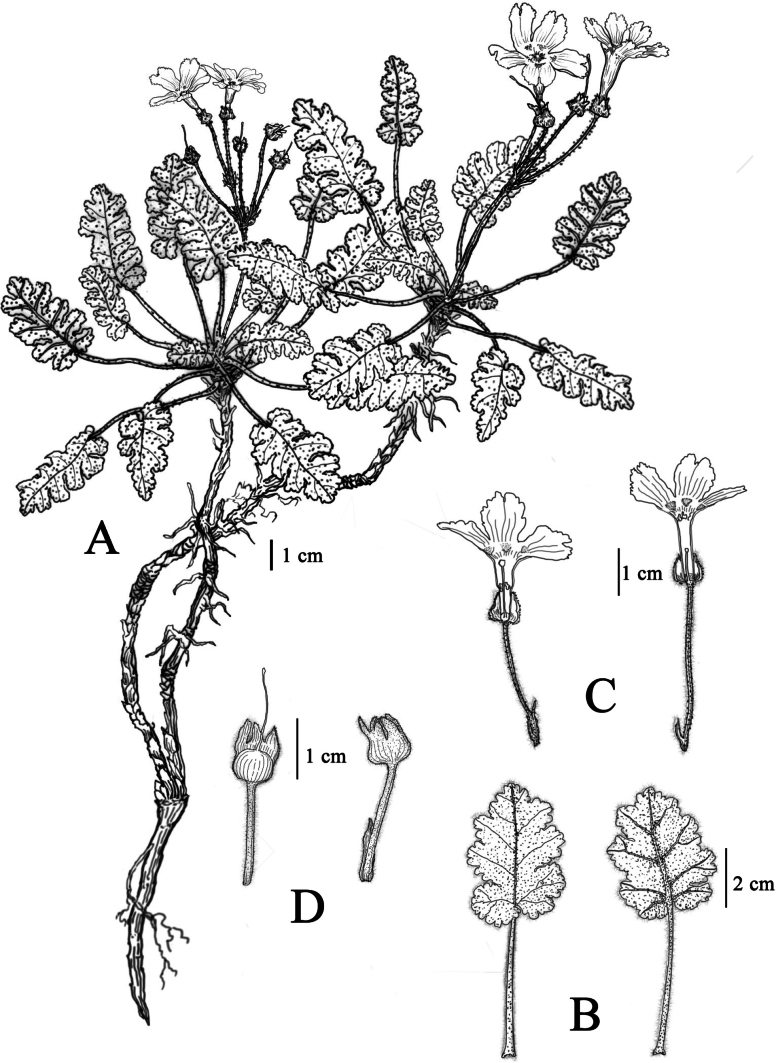
*Primulajiangyouensis* sp. nov. **A.** Habit; **B.** Leaves, left: upper surface, right: lower surface; **C.** Flower, left: pin flower, right: thrum flower; **D.** Calyx and young fruit. Drawn by Ms. Xiang-Li Wu.

**Figure 3.**
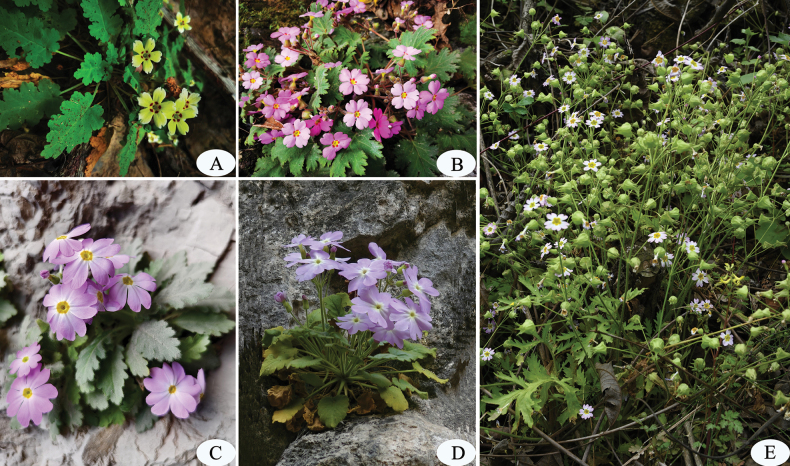
*Primulajiangyouensis* and three of its allies. **A.***P.jiangyouensis*; **B.***P.sinensis*; **C.***P.xingshanensis*; **D.***P.rupestris*; **E.***P.filchnerae*. Photos **A–D**: Z.K.Wu, E: J.L.Gu.

**Figure 4.**
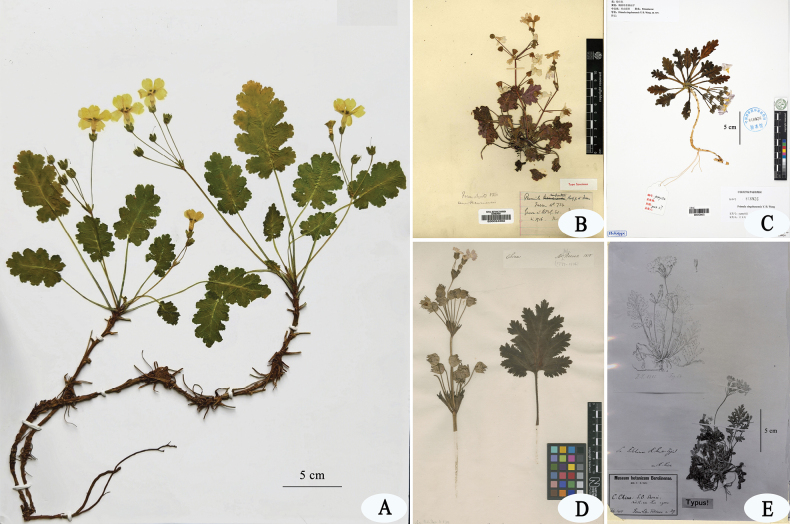
Type specimens of *Primulajiangyouensis* and three of its allies. **A.** Holotype of *P.jiangyouensis* (GJL384, KUN!); **B.** Holotype of *P.rupestris* (Farrer 734, E!); **C.** Holotype of *P.xingshanensis* (YCMY 152, IBSC!); **D.** Lectotype of *P.sinensis* (BM barcode BM000996827); **E.** Type of *P.filchnerae* (photo, Filchner 37, E00024341).

The new species is most similar to *P.sinensis*, *P.rupestris*, and *P.xingshanensis*, sharing hairs that covered the leaves and stems, lobed leaf blade, distinctly petiole and distinctively broad and flat-bottomed calyx. However, the new species is distinguished from the latter three mainly by its branched and stout rhizomes usually up to 40 cm, yellow corollas with a distinct fan-shaped reddish-brown blotch at the base of lobes, and short glandular hair on aboveground parts (Figs 1–4). The main morphological distinctions between *P.jiangyouensis*, *P.sinensis*, *P.rupestris*, and *P.xingshanensis* are summarized in Table 1.

**Table 1.** Morphological comparison between *P.jiangyouensis*, *P.rupestris, P.xingshanensis* and *P.sinensis*.

Characters	*P.jiangyouensis*	*P.rupestris*	*P.xingshanensis*	*P.sinensis*
Rhizomes	Stout, usually with 2–8 branches, up to 40 cm long, with numerous dry old leaves at the apex	Comparatively stout, occasionally branched, up to 15 cm long, with numerous dry old leaves at the apex	Comparatively stout, occasionally branched, up to 25 cm long, with numerous dry old leaves at the apex	Comparatively stout, unbranched, up to 10 cm, without dry old leaves at the apex
Petiole	pale red or green, 4–16 cm long	green, 4–13 cm long	green, 1.0–5 cm long	purplish, 4–15 cm long
Leaf blade	oblong-ovate to ovate-orbicular	ovate-rotund to ovate-elliptic	obovate-spatulate to spatulate	ovate to subrotund
Hair of the aboveground parts	short glandular hairs	short glandular hairs	long multicellular white hairs	long multicellular white hairs
Corolla	yellow, with a fan-shaped reddish-brown blotch at the base of lobes	white, pale lilac or rose, with a yellowish-green eye at the throat	pink or pale lilac, with a yellow eye at the throat	white, pink, rose or purple with a yellow eye at the throat, sometimes with a rose blotch at the base of lobes
Flowering time	November to February of the following year	late December to February of the following year	February to March	January to March

#### Type.

China • Sichuan: Mianyang, Jiangyou County, Yongshen town, Jingtai village. 32°0'31"N, 104°53'26"E, 1070 m alt., 12 November 2022 (fl.), *Jiulin Gu*, GJL384 (***holotype***: KUN!; ***isotype***: KUN!).

#### Description.

Perennial herb, the entire aboveground parts densely covered with short glandular hairs; roots purplish-brown and pale purple, few in number, fibrous and brittle; rhizome woody, stout and elongated, up to 40 cm long and 1 cm thick, usually with 2–8 branches, bearing numerous persistent dry leaf sheath from base to apex. ***Leaves*** mostly clustered at the apex of the rhizome, rosette, including petiole 8–24 cm long; petiole 4–16 cm long, densely covered with short glandular hairs, base expanded, pale red or green, brittle when fresh; leaf blade oblong-ovate to ovate-orbicular, 4–8 cm long, 2–6 cm wide, apex obtuse-rounded, base truncate and oblique, margin pinnately 4–6-lobed, parted up to 1/2 or more of the blade, lobes oblong, each side with 3–12 obtuse notched teeth, slightly thick when fresh, papery when dry, mid-rib prominent, lateral veins 5–7 pairs, slightly concave adaxially and densely covered with short glandular hairs, raised abaxially and densely glandular-hairy along veins. ***Scape*** 5–35 cm tall, green; umbels 1–5-whorled, each whorl with 2–11 flowers. ***Bracts*** lanceolate, entire, 5–10 mm long, acute at apex, glandular-hairy. ***Pedicels*** 2–6 cm long, glandular-hairy. ***Calyx*** 6–9 mm long, base inflated and hemispherical, 4–6 mm in diameter, enlarging in fruit to 8–12 mm in diameter, mostly 5-lobed (rarely 4 or 6), cut up to half its length, lobes triangular, densely glandular-hairy on both surfaces. ***Corolla*** yellow, glandular-hairy outside, limb 1.5–3.2 cm in diameter, throat yellow, without an annular appendage; lobes broadly obovate, apex bifid, margin entire or lacerate, with a fan-shaped reddish-brown blotch at the base. ***Flower*** heterostylous, Pin flowers: corolla tube 14–19 mm long, stamens inserted near the middle of the tube, style 10–18 mm long, nearly reaching or slightly exceeding the throat; thrum flowers: corolla tube 15–19 mm long, stamens inserted 10–15 mm from base of corolla tube, style 4–7 mm long. ***Capsule*** ovoid, 5–10 mm in diameter, shorter than the calyx. ***Seeds*** globose or ovoid, about 1.0 mm in diameter, finely vesicular-surfaced.

#### Distribution and habitat.

**Map 1.**
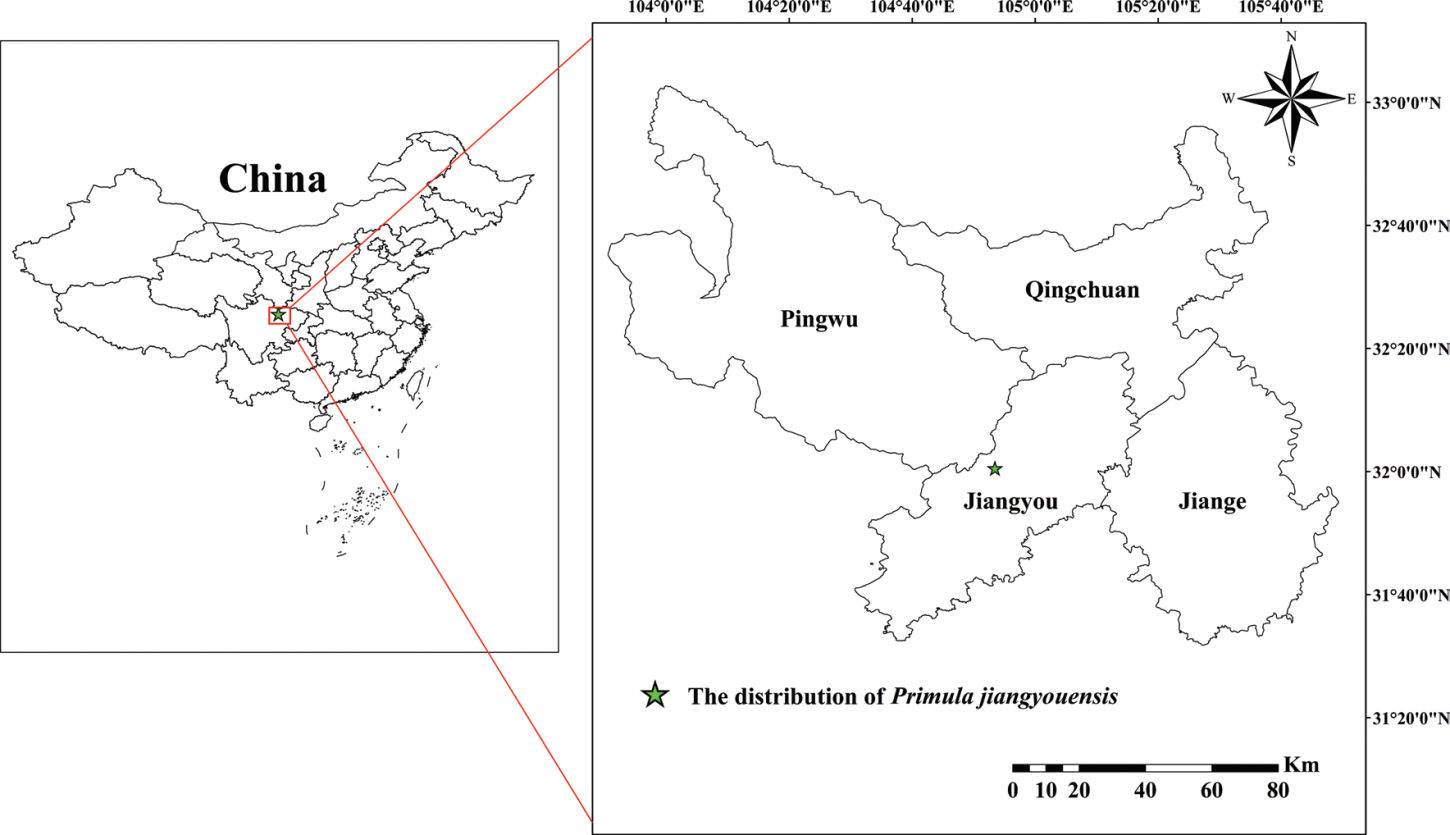
Location of the distribution of *Primulajiangyouensis* in Jiangyou, Sichuan.

The new species is currently known from the type locality near Jingtai village, Cangwangzhai Scenic Area, Yongshen town, Jiangyou County, Sichuan Province, China, and is mostly found on shady ledges of limestone cliffs, at an altitude of 1070–1550 m (Fig. 1, Map 1).

#### Phenology.

The species was observed to flower from November to February of the following year, fruiting from March to April.

#### Etymology.

The specific epithet of the new species is taken from the Chinese Pinyin “Jiangyou”, the name of the county in northern Sichuan, China, where the type specimen was collected (Map 1).

#### Vernacular name.

Chinese mandarin: *jiang you bao chun* (江油报春).

#### Provisional conservation status.

This species exhibits a highly restricted distribution range, currently documented solely from the type locality within the Cangwangzhai Scenic Area. Field investigations have identified three spatially proximate but ecologically isolated subpopulations occupying distinct microhabitats. These subpopulations occur on fragmented shady limestone cliffs at varying elevations (ranging from 1070 to 1550 m a.s.l.). Population viability analysis indicates that the total number of mature individuals across all subpopulations is critically less than 250. Longitudinal monitoring data (December 2023 and April 2024) reveal progressive contraction in the area of occupancy (AOO) and continuing degradation of habitat quality. The principal threatening processes include: (1) climate change-induced alteration of microclimatic conditions (2) accelerated habitat fragmentation from geological processes; (3) edge effects exacerbating population isolation. Applying the IUCN Red List Categories and Criteria (Version 16.0, 2024), our assessment demonstrates that *P.jiangyouensis* meets the threshold for Critically Endangered (CR) status under criterion B1ab (i,ii,iii), based on: extent of occurrence (EOO) <100 km^2^ (B1), severely fragmented habitat (a), with continuing decline (b) in extent of occurrence (i), area of occupancy (ii) and area, extent and/or quality of habitat (iii).

#### Additional specimens examined

**(*paratypes*).** The same locality as holotype, 21 November 2022, *Jiulin Gu* & *Huadong Wang*, GJL385 (KUN!); 28 December 2023, *Zhikun Wu*, ZKWu 2023305 (KUN!), 27 April 2024, *Zhikun Wu*, ZKWu 2024060 (KUN!).

## ﻿Discussion

PrimulaSectionAuganthus represents a small and morphologically distinct group within the genus *Primula*. Historically, its distinctive calyx morphology led to its treatment at generic rank under three heterotypic names: *Auganthus* Link, *Oscaria* Lilja, and *Primulidium* Spach. Pax subsequently established this group as SectionSinenses Pax within *Primula*, initially circumscribing it to include several species distributed predominantly in western China and the eastern Himalayas (Pax 1889). Later taxonomic revisions reassigned these species among various sections of subgenus Auganthus (Duby) Wendelbo, with calyx morphology remaining the primary diagnostic character. P.Sect.Auganthus is most readily distinguished by its unique calyx structure: the broadly inflated basal portion contrasts with the constricted upper portion that tightly adheres to the corolla tube, which is not observed in other *Primula* sections. However, current classifications of subgenus Auganthus sections demonstrate polyphyly, with inconsistent placements among taxonomists based on differing morphological interpretations. Smith and Fletcher recognized two species (*P.sinensis* and *P.rupestris*) in this section, while assigning *P.filchnerae* to P.Sect.Pinnatae due to its pinnatifid leaves (Smith and Fletcher 1946). “Flora Reipublicae Popularis Sinicae” treated *P.rupestris* as conspecific with *P.sinensis*, reducing the section to just two taxa (Hu 1990). Halda (1992) maintained this classification in his monograph, while Hu and Kelso (1996) reinstated *P.rupestris* to specific rank in “Flora Of China”. Richards (2002) followed Smith and Fletcher’s treatment of *P.filchnerae*. Phylogenetic analysis based on chloroplast genome data revealed that *P.filchnerae* clusters with *P.sinensis*, distinct from other species of subgenus Auganthus (Sun et al.2019), supporting its placement in P.Sect.Auganthus based on calyx morphology. Wang et al. (2023) subsequently described *P.xingshanensis* from Hubei Province, expanding the section to four species. The newly discovered *P.jiangyouensis* unequivocally belongs to this section based on its diagnostic inflated calyx. However, it exhibits several distinct traits: exceptionally elongated rhizomes, yellow corollas with a fan-shaped reddish-brown blotch at the base of lobes, and short glandular hair on aerial parts. Field studies indicate P.Sect.Auganthus species predominantly occur on karst limestone formations at the Sichuan-Shaanxi-Hubei provincial junction. The fragmented nature of these edaphic islands likely promoted allopatric speciation through geographic isolation and local adaptation. The discovery of *P.jiangyouensis* provides critical insights into the study of evolutionary radiation and species differentiation within this section.

### ﻿Key to the species of PrimulasectionAuganthus

From the distribution of five species, all are endemic to China. To facilitate the identification of these five species, a key is constructed as follows (adapted from Wang et al. 2023):

**Table:** 

1	Leaves pinnately lobed to midvein	***P.filchnerae***
–	Leaves pinnately or palmately lobed, lobes not reaching midvein	**2**
2	Old leaves falling off, petiole purplish	***P.sinensis***
–	Old leaves remaining, petiole green	**3**
3	Leaf blade base cuneate, with long multicellular white hairs	***P.xingshanensis***
–	Leaf blade base cordate to sub-truncate, with short glandular hairs	**4**
4	Rhizomes occasionally branched, up to 15 cm long, corolla white, pale lilac or rose	***P.rupestris***
–	Rhizomes branched, up to 40 cm long, corolla yellow	***P.jiangyouensis***

## Supplementary Material

XML Treatment for Primula jiangyouensis

## ﻿Additional information

### Use of AI

No use of AI was reported.

### Author ORCIDs

Jiu-Lin Gu https://orcid.org/0009-0007-7557-0445

Hua-dong Wang https://orcid.org/0009-0008-4943-1035

Hong-qiang Lin https://orcid.org/0000-0001-8152-4911

Xiao-qi Jiang https://orcid.org/0000-0002-2101-6541

Tian Shuai https://orcid.org/0009-0009-7314-8026

Zhi-kun Wu https://orcid.org/0000-0002-4583-7782

### Data availability

All of the data that support the findings of this study are available in the main text.
